# Modified Frailty Index and Postoperative Complications in Seniors Undergoing Elective Aesthetic Surgery

**DOI:** 10.1093/asjof/ojag138.002

**Published:** 2026-07-24

**Authors:** Anandhini D Narayanan, Alexis K Gursky, Hailey P Wyatt, Sergio A Segrera, Eduardo D Rodriguez

## Abstract

**Purpose:**

Elderly individuals, aged 65 years and older, represent a growing segment of patients seeking aesthetic surgery. However, preoperative risk assessment in this population still relies mainly on chronological age, ASA class, and BMI, all of which only partially reflect physiologic reserve. Existing safety data in elderly aesthetic patients have focused largely on facial procedures or are more than a decade old, and do not address contemporary breast and body procedure that are frequently performed in ambulatory or short-stay settings. This study evaluated whether the 5- item modified frailty index (mFI-5) identifies patients older than 65 years who are at higher 30- day risk after elective aesthetic breast and body procedures, beyond age, ASA, and BMI.

**Methods:**

We performed a retrospective cohort study using the American College of Surgeons National Surgical Quality Improvement Program (ACS-NSQIP) from 2009-2023. We identified elective aesthetic breast and body procedures by CPT code and excluded reconstructive/oncologic, postbariatric, emergency, and combined cases to approximate an aesthetic surgical population. Two outcomes were evaluated: (1) wound complication, defined as a composite of superficial, deep, or organ-space surgical site infection or wound dehiscence; and (2) any 30-day complication, defined as wound events plus medical complications, unplanned reoperation, or mortality. We first described overall 30-day complication rates for the entire aesthetic cohort to provide context for practicing surgeons. We then restricted all inferential analyses – bivariate comparisons, univariate logistic regression, and multivariable logistic regression – to patients aged 65 years and older. For each patient, mFI-5 was calculated from NSQIP variables (diabetes, hypertension requiring medication, history of congestive heart failure, chronic obstructive pulmonary disease, and partially or totally dependent functional status) and prescribed as a dichotomous variable, mFI-5 > 2 vs.

**Results:**

We identified 111,173 patients who underwent elective aesthetic breast or body procedures during the study period. In the overall cohort, the 30-day rate of any complication was 6.5%, and the wound complication rate was 3.9%, confirming that complications after aesthetic breast/body surgery are uncommon in NSQIP-captured settings. Of these, 7,703 (6.9%) were aged 65 years or older; 6,040 underwent breast procedures and 1,663 underwent body procedures. Older patients were more likely to have higher frailty scores: 13.8% of seniors had mFI-5 > 2, compared with 3.2% in the entire cohort (p<0.001).

Within the > 65 group, mFI-5 clearly stratified risk. Patients with mFI-5 > 2 had higher crude rates of any complication (13.4% vs. 7.2%) and wound complication (7.2% vs. 3.9%), both p 2 accounted for 23.0% of all complications and 22.8% of all wound complications, indicating a concentrated high-risk subgroup within an otherwise relatively fit, clinically preselected elderly population. Patients with mFI-5 > 2 were also more likely to undergo body procedures more than those with mFI-5 < 2 (39.1% vs. 18.8%, p<0.001), suggesting that procedure mix alone could not explain the observed difference in complication burden.

In multivariable logistic regression restricted to patients > 65 years and adjusting for known surgical risk factors, mFI-5 > 2 remained independently associated with any 30-day complication (OR 1.32, 95% CI 1.03-1.70; p=0.028). BMI retained an important role: each 5 kg/m2 increase in BMI was associated with higher odds of any complication (OR 1.28, 95% CI 1.16-1.34) and of wound complications (OR 1.34, 95% CI 1.22-1.47), both p<0.001. These findings suggest that, in older aesthetic patients, frailty and BMI represent two distinct and additive signals.

**Conclusion:**

In a large, contemporary national sample, 30-day complications after aesthetic breast and body surgery were uncommon overall. However, among adults aged 65 years and older, mFI-5 > 2 identified a small but disproportionately high-risk subgroup, even after adjustment for age, ASA class, BMI, and procedure category.

A common argument is that aesthetic surgeons already self-select healthier older adults, and therefore formal frailty scoring is unnecessary. Our data support that clinicians are indeed selecting relatively fit seniors – only 6.8% of all aesthetic breast/body patients were 65 or older – yet even in this preselected group, a brief 5-item frailty screen identified a small subset (13.8%) that generated nearly one quarter of all complications. This indicates that routine clinical judgement does not fully capture functional reserve. Because the mFI-5 uses elements already available to most surgeons (diabetes, blood pressure treatment, cardiopulmonary history, dependence), it can be incorporated at consultation and can inform several decisions: (1) more explicit risk counseling for seniors with mFI-5 > 2; (2) targeted preoperative optimization of cardiometabolic and pulmonary status; and (3) selection of higher-acuity ambulatory or shortstay settings.

